# Kidney Function Is Not Related to Brain Amyloid Burden on PET Imaging in The 90+ Study Cohort

**DOI:** 10.3389/fmed.2021.671945

**Published:** 2021-09-20

**Authors:** Wei Ling Lau, Mark Fisher, Evan Fletcher, Charles DeCarli, Hayden Troutt, María M. Corrada, Claudia Kawas, Annlia Paganini-Hill

**Affiliations:** ^1^Division of Nephrology and Hypertension, University of California, Irvine School of Medicine, Orange, CA, United States; ^2^Department of Neurology, University of California, Irvine School of Medicine, Irvine, CA, United States; ^3^Department of Anatomy & Neurobiology, University of California, Irvine School of Medicine, Irvine, CA, United States; ^4^Department of Pathology & Laboratory Medicine, University of California, Irvine School of Medicine, Irvine, CA, United States; ^5^Department of Neurology, Center for Neuroscience, University of California, Davis, Davis, CA, United States; ^6^Institute for Memory Impairments and Neurological Disorders, University of California, Irvine, Irvine, CA, United States; ^7^Department of Epidemiology, University of California, Irvine School of Medicine, Irvine, CA, United States; ^8^Department of Neurobiology and Behavior, University of California, Irvine School of Medicine, Irvine, CA, United States

**Keywords:** chronic kidney disease, cystatin C, amyloid PET, aging, cognitive decline and dementia

## Abstract

Cognitive decline is common in chronic kidney disease (CKD). While the evidence of vascular cognitive impairment in this population is robust, the role of Alzheimer's pathology is unknown. We evaluated serum cystatin C-estimated glomerular filtration rate (eGFR), brain amyloid-β positron emission tomography (PET) imaging, and cognitive function in 166 participants from The 90+ Study. Mean age was 93 years (range 90-107) and 101 (61%) were women; 107 participants had normal cognitive status while 59 participants had cognitive impairment no dementia (CIND) or dementia. Mean ± standard deviation cystatin C was 1.59 ± 0.54 mg/L with eGFR 40.7 ± 18.7 ml/min/1.73m^2^. Higher amyloid-β burden was associated with dementia, but not with age, diabetes, hypertension, or cardiovascular disease. We found no association between brain amyloid-β burden and cystatin C eGFR. We previously reported that kidney function was associated with cognition and cerebral microbleeds in the same cohort of oldest-old adults (90+ years old). Collectively, these findings suggest that microvascular rather than Alzheimer's pathology drives CKD-associated cognitive dysfunction in this population.

## Introduction

Chronic kidney disease (CKD) is increasingly recognized as an independent risk factor for cerebrovascular disease and cognitive decline (1–3). This association appears to be robust even with advanced age: in an oldest-old cohort of community-dwelling adults aged 90+ years, our group recently reported a significant association between CKD and incident dementia as well as infratentorial cerebral microbleeds (4). Microvascular disease in CKD includes blood-brain barrier dysfunction, cerebral microbleeds, gray matter atrophy, and arteriolar neuropathology; it is driven by factors such as chronic inflammation, uremic toxins and impaired cerebral blood flow autoregulation (5–7).

Cystatin C, a low molecular weight (13 kDa) protease inhibitor produced by all nucleated cells in the body, is freely filtered through the glomeruli and degraded by proximal tubular cells (8). Cystatin C accumulates in CKD and is a more valid estimation of kidney function (estimated glomerular filtration rate, eGFR) than creatinine in older individuals since it is not affected by diet or muscle mass (9, 10). Compared with creatinine-based eGFR, cystatin C-based eGFR is a stronger predictor of mortality and frailty outcomes in elderly cohorts (11, 12).

While cerebral small vessel disease is a well-established phenomenon in CKD (7, 13), the potential relationship between CKD and brain amyloid-β deposits is less clear. Of note, cystatin C has been reported to co-localize with amyloid-β, which aggregates in the hippocampus and entorhinal cortex in individuals suffering from Alzheimer's disease (14). In addition, a common polymorphism of the cystatin C gene has been linked to risk of Alzheimer's disease (15, 16). We therefore hypothesized that higher blood cystatin C (more advanced CKD) would be associated with increased brain amyloid-β burden.

Imaging of amyloid-β densities through positron emission tomography (PET) is an emerging tool for non-invasive monitoring of amyloid deposition (17). Amyloid-β PET is being explored for diagnosis of Alzheimer's pathology and may have utility in tracking treatment response (18, 19). This imaging tool has previously been explored in a small cohort of 90+ year-olds (17). In the current study we examined the association between amyloid-β imaging with (a) cognitive function and (b) cystatin C, in an oldest-old cohort of community-dwelling adults.

## Materials and Methods

We report results from a subset of participants of The 90+ Study, an ongoing longitudinal study of aging and dementia in people aged 90 or older (4, 20, 21). Participants of The 90+ Study were recruited from two groups: (1) survivors of the Leisure World Cohort Study (20), an epidemiological health study established in the 1980s of the residents of Leisure World, a retirement community in Orange County, California, who were aged 90 or older on or after January 1, 2003, when enrollment into The 90+ Study commenced, and (2) 90+ year-old residents of Orange County, California, who lived within a 2-h drive of the study location, and joined the study through open recruitment (21). Participants self-reported their birthdate, education, and medical history. The Institutional Review Board (IRB) of the University of California, Irvine (UC Irvine) approved this study.

### Amyloid PET Scan

Imaging of amyloid-β densities through PET scan is an emerging tool for non-invasive monitoring of amyloid deposition (17). Between 2009 and 2020, 308 participants underwent a 10-min PET scan at ~50 min after injection of 370 MBq of Florbetapir F18 (17). After quality control and alignment of all native PET images to a standard template space, standard uptake value ratios (SUVR) were computed using an eroded cerebral white matter region reference. To obtain brain indices of amyloid-β deposition we used a statistically defined region of interest (statROI) consisting of precuneus and posterior cingulate cortices. This region was chosen because the distribution of mean SUVR produced a maximal separation of normal from cognitively impaired individuals.

### Cystatin C and eGFR

Blood draw for cystatin C was added to the IRB protocol in July 2014, was collected at the time of the PET scan until April 2017. Since May 2017 cystatin C has been measured at the time of a regularly scheduled follow-up visit. Cystatin C taken at or near the time of the PET scan was available for 166 of the 308 participants. Serum cystatin C was measured using the Latex Enhanced Immunoturbidimetric Method by the Pathology and Laboratory Services of the UC Irvine Medical Center. Estimated glomerular filtration rate (eGFR) was calculated from cystatin C based on the Chronic Kidney Disease-Epidemiology Collaboration (CKD-EPI) equation which accounts for age and sex (22). The blood sample taken closest to the PET scan was selected for analysis.

### Neuropsychological Examination and Cognitive Status Evaluation

Participants were seen every 6 months and given a standard battery of 10 neuropsychological tests indexing multiple cognitive domains and including the Modified Mini-Mental State Examination (3MS), by trained and certified psychometrists. Participants also underwent a neurological exam by neurological examiners (trained physicians or nurse practitioners) to determine cognitive status (normal, cognitive impairment no dementia [CIND], or dementia) (4, 23). During this visit, medical history was also updated. The visit closest to the PET scan was selected for analysis.

### Data Analysis

Means and standard deviations (SD) of PET statROI were calculated for demographic, medical history, and cognitive status categories. Differences in means were tested using *t*-tests and analysis of variance (with *post-hoc* analysis for multiple comparison tests of means). Spearman rank correlation and partial correlation coefficients were calculated for age, eGFR and PET statROI. Spearman rank correlation coefficient between PET statROI and CKD stage was also calculated. With 166 participants, the study was powered to detect a significant correlation of >0.22. All statistical analyses were performed using SAS software version 9.4 for Windows (SAS Institute Inc., Cary, NC).

## Results

Of the 308 participants with an amyloid PET scan, cystatin C measurement was available in 166 participants. Of these 166, 101 (61%) were women and average age was 93 years. The majority of participants (*n* = 94) completed blood-draw for cystatin C and the amyloid PET scan on the same day; all but 10 participants completed amyloid PET scan within 90 days of cystatin C measurement. Cystatin C ranged 0.72-3.02 mg/L (mean 1.59, SD 0.54) and eGFR ranged 14 to 92 ml/min/1.73 m^2^ (mean 40.7, SD 18.7). The number of participants by CKD stage were: CKD stage 2, *n* = 29 (17%); CKD stage 3a, *n* = 27 (16%); CKD stage 3b, *n* = 54 (33%); CKD stage 4, *n* = 53 (32%); and CKD stage 5, *n* = 3 (2%). PET statROI ranged 0.59-0.93 (mean 0.76, SD = 0.07).

Table 1 gives the mean ± SD of the PET statROI by the participants' characteristics in the 166 participants with cystatin C measurements. Neither sex, education, smoking nor any of the medical history variables was related to PET statROI. Examining the larger cohort of 308 participants with PET imaging, participants with vs. without cystatin C measurement (166 vs. 142 participants) differed only on coronary artery disease (9 vs. 18%, *p* = 0.02; data not shown). The two groups did not differ on body mass index nor thyroid stimulating hormone, factors which can modify cystatin C levels (24, 25). We had no information on inflammatory markers such as C reactive protein.

**Table 1:** Mean + standard deviation (SD) of brain amyloid-β burden measured *via* positron emission tomography statistically defined region of interest scores (PET statROI) by participants' characteristics, The 90+ Study.

	**Category**	**Number**	**Mean + SD (range)**	***P*-value^†^**
All		166	0.76 + 0.07 (0.59-0.93)	
Sex	Male	65	0.75 + 0.07 (0.59-0.93)	0.27
	Female	101	0.76 + 0.07 (0.60-0.92)	
Education: college grad	No	77	0.75 + 0.07 (0.60-0.91)	0.17
	Yes	89	0.77 + 0.07 (0.59-0.93)	
Smoking history	Never	80	0.77 + 0.07 (0.59-0.93)	0.10
	Past	85	0.75 + 0.07 (0.60-0.92)	
**Medical history**				
High blood pressure	No	75	0.75 + 0.07 (0.59-0.92)	0.32
	Yes	91	0.76 + 0.07 (0.62-0.93)	
Diabetes	No	148	0.76 + 0.07 (0.59-0.93)	0.52
	Yes	18	0.75 + 0.09 (0.62-0.90)	
Coronary artery disease	No	151	0.76 + 0.07 (0.59-0.92)	0.87
	Yes	15	0.76 + 0.07 (0.68-0.93)	
Heart attack	No	155	0.76 + 0.07 (0.59-0.93)	0.69
	Yes	11	0.77 + 0.09 (0.62-0.90)	
Heart valve disease	No	159	0.76 + 0.07 (0.59-0.93)	0.39
	Yes	7	0.74 + 0.08 (0.65-0.90)	
Congestive heart failure	No	154	0.76 + 0.07 (0.59-0.93)	0.32
	Yes	12	0.74 + 0.08 (0.60-0.92)	
Stroke	No	150	0.76 + 0.07 (0.59-0.93)	0.45
	Yes	16	0.75 + 0.06 (0.66-0.86)	
TIA	No	137	0.76 + 0.07 (0.60-0.92)	0.14
	Yes	29	0.78 + 0.08 (0.59-0.93)	
Any of above	No	44	0.77 + 0.07 (0.64-0.92)	0.52
	Yes	122	0.76 + 0.07 (0.59-0.93)	
Depression	No	151	0.76 + 0.07 (0.59-0.93)	0.59
	Yes	15	0.77 + 0.07 (0.68-0.86)	
Cognitive status	Normal	107	0.75 + 0.07 (0.60-0.92)	**0.004**
	Cognitive Impairment No Dementia (CIND)	52	0.76 + 0.08 (0.59-0.93)	
	Dementia	7	0.84 + 0.06 (0.71-0.89)	

† *For test for difference in mean statROI between groups. Bold values were statistically significant P < 0.05*.

**Figure 1:**
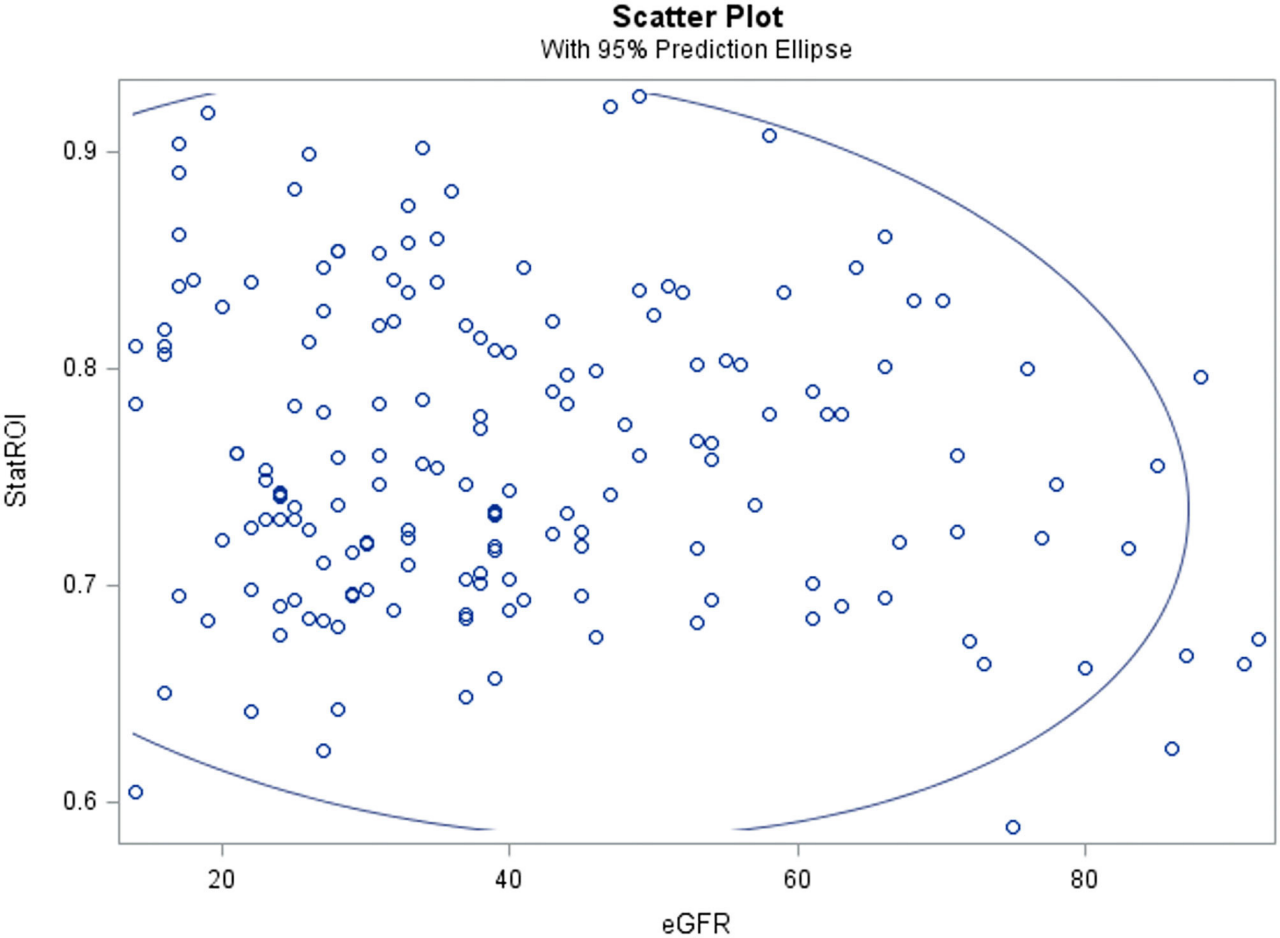
Brain amyloid-β burden assessed non-invasively on positron emission tomography (statistically defined region of interest score, statROI) was not correlated with estimated glomerular filtration rate (eGFR) in a cohort of community-dwelling 90+ year-olds.

Mean statROI was significantly higher in those with dementia (0.84) than in those with normal cognition (0.75) or CIND (0.76), *p* = 0.004. In subgroup analysis of the 94 participants who had same-day cystatin C blood-draw and amyloid PET scan, the association with cognitive impairment remained consistent and significant (*p* = 0.01). PET statROI was not correlated with cystatin C (r = 0.09), eGFR (−0.09) or age (r = 0.12) (Table 2 and Figure 1) or with CKD stage (r = 0.05) (Table 3).

**Table 2:** Spearman correlation and partial correlation coefficients of estimated glomerular filtration rate (eGFR), brain amyloid-β positron emission tomography statistically defined region of interest scores (PET statROI), and age (years), The 90+ Study.

	**Mean + SD (range)**	**Spearman correlation (and partial correlation) coefficients**		
		eGFR	StatROI	Age
eGFR	40.7 + 18.7 (14–92)	1.00	−0.09 (−0.06)	−0.33 (−0.32)^†^
StatROI	0.76 + 0.07 (0.59-0.93)		1.00	0.12 (0.10)
Age	93 + 2.8 (90-107)			1.00

† *p < 0.0001 for correlation of eGFR and age*.

**Table 3:** Mean + standard deviation (SD) of PET StatROI by CKD stage, The 90+ Study.

**CKD stage**	**Mean + SD (range)**	**Number**
2	0.73 + 0.07 (0.59-0.86)	29
3a	0.78 + 0.07 (0.68-0.93)	27
3b	0.76 + 0.06 (0.65-0.90)	54
4	0.76 + 0.08 (0.62-0.92)	53
5	0.73 + 0.11 (0.60-0.81)	3
Spearman rank correlation = 0.05, *p* = 0.54		

## Discussion/Conclusion

The current study demonstrates a significant association between brain amyloid-β burden (measured on PET scan) and cognitive impairment in an oldest-old cohort from The 90+ Study, but no association between cystatin C eGFR or CKD stage and brain amyloid-β burden. We have previously reported significant association between CKD, indices of cerebral microvascular disease, and cognitive decline in the same cohort (4). Collectively, our work suggests that decreased kidney function and brain amyloid-β burden impact cognition *via* different pathophysiologic pathways.

Although higher serum amyloid-β levels have been reported in CKD patients (26), this may be due to decreased renal clearance of amyloid-β (27). Our current study in a non-dialysis elderly cohort is consistent with the report by Reusche et al. of a post-mortem analysis from 50 patients with end-stage kidney failure on chronic hemodialysis (28). No increase in Alzheimer's disease morphology, compared with age-matched controls, was observed (28). In the current study we found no relationship between CKD and brain amyloid-β burden, assessed non-invasively. Similarly, in a French cohort of community-dwellers >70 years old where eGFR was relatively preserved (median eGFR 73 with interquartile range 60-84 ml/min/1.73 m^2^), CKD was associated with cognitive decline over time but was not associated with imaging features of Alzheimer's disease (cortical amyloid-β and hippocampal atrophy) (29). A strength of the current study is the use of cystatin C eGFR measurements, which are not modified by diet or muscle mass and thus are more valid than creatinine-based eGFR in elderly individuals (9, 10).

In contrast to the lack of association with Alzheimer's-associated pathology, prior work by us and others have shown an association between CKD and cerebral microvascular disease (4, 6, 13, 30, 31). CKD-associated cerebral small vessel disease includes microbleeds, microinfarcts, lacunes, white matter or global atrophy, and arteriolosclerosis (5). In a prior analysis of The 90+ Study cohort, lower kidney function correlated with impaired global cognition, executive function, and visual-spatial ability; infratentorial microbleeds; and lower gray matter volume (4). Risk of incident dementia in the highest cystatin C tertile was 3.81 (adjusted for age, sex, education, and comorbid conditions) and was attenuated when microbleeds were included in the risk model, suggesting that the impact of CKD on cognitive dysfunction is partly mediated by microbleeds (4). In 2,526 participants from the population-based Rotterdam Study, lower cystatin C-based eGFR was associated with a higher prevalence of lacunes and larger white matter lesion volume (31). It is important to note that certain risk factors for Alzheimer's disease are prominent in CKD, including vascular dysfunction (27) and elevated serum homocysteine (32–34). Given these shared risk factors, studies that correlate CKD with risk of Alzheimer's disease require careful interpretation (27, 35). In the current study, evaluation of amyloid-β which is a pathological substrate for Alzheimer's was not correlated with kidney function.

We acknowledge several study limitations. The 90+ Study provides robust longitudinal data on individuals aged 90 years and older, but as the majority of participants are white, highly educated and moderately affluent the results may not be generalizable to other population groups. We cannot rule out survival bias, as community-dwelling individuals in The 90+ Study have relatively good cardiovascular health and do not have advanced CKD. Our study may be underpowered to detect an association between CKD and brain amyloid-β burden. Finally, although most participants (*n* = 94) had cystatin C measurement and amyloid PET imaging on the same day, the interval between PET imaging and blood collection for cystatin C did vary. However, subgroup analysis limited to participants who had same-day measurement of cystatin C and amyloid-β PET imaging demonstrated a consistent positive association between higher statROI with worse cognitive impairment, suggesting that the overall group analyses remain representative.

In summary, cystatin C-estimated kidney function was not associated with brain amyloid-β burden in adults > 90 years of age. Prior work by us and others have demonstrated an independent association between CKD with cognition (2–4, 36, 37) and cerebral microvascular disease (4, 6, 30, 31). These findings suggest that cognitive impairment in the CKD population largely reflects vascular rather than amyloid-β pathology.

## Data Availability Statement

The original contributions presented in the study are included in the article; further inquiries can be directed to the corresponding author.

## Ethics Statement

The studies involving human participants were reviewed and approved by University of California, Irvine IRB. The participants provided their written informed consent to participate in this study.

## Author Contributions

WL, MF, HT, and AP-H drafted the initial manuscript. EF, CD, and AP-H did data analysis. AP-H, CK, and MC are PIs for the longitudinal patient cohort and oversee data extraction. All authors provided edits and approved the final version of the manuscript.

## Funding

Research reported in this publication was supported by the National Institute on Aging of the National Institutes of Health under award number R01AG02105 (AP-H, CK, and MC). Approximately $2,205,309 (100%) of federal direct funds supported this project. Publication costs were covered by unrestricted, nonfederal funds from the Division of Nephrology, Department of Medicine at University of California, Irvine. The content is solely the responsibility of the authors and does not necessarily represent the official views of the National Institutes of Health.

## Conflict of Interest

The authors declare that the research was conducted in the absence of any commercial or financial relationships that could be construed as a potential conflict of interest.

## Publisher's Note

All claims expressed in this article are solely those of the authors and do not necessarily represent those of their affiliated organizations, or those of the publisher, the editors and the reviewers. Any product that may be evaluated in this article, or claim that may be made by its manufacturer, is not guaranteed or endorsed by the publisher.
